# Beiträge kommunaler Planung für mehr Ernährungssicherheit in deutschen Städten

**DOI:** 10.1007/s00548-023-00840-7

**Published:** 2023-03-15

**Authors:** Hanna Augustin, Marit Rosol

**Affiliations:** 1 Die Senatorin für Klimaschutz, Umwelt, Mobilität, Stadtentwicklung und Wohnungsbau, Contrescarpe 27, 28195 Bremen, Deutschland; 2 Institut für Geographie und Geologie, Julius-Maximilians-Universität Würzburg, Am Hubland, 97074 Würzburg, Deutschland

**Keywords:** Zugang zu Lebensmitteln, Armut, Einzelhandelssteuerung, Soziale Ungleichheit, Agri-Food Geographien, Access to food, Household food insecurity, Municipal retail governance, Social inequality, Agri-food geographies

## Abstract

Zur Einzelhandelssteuerung entwickeln viele deutsche Kommunen Einzelhandelskonzepte. Eine Bestandsaufnahme der Lebensmittelversorgung gehört dabei inzwischen zu den Standardinstrumenten. Gemeinhin wird dabei ein distanzbasierter Indikator verwendet, der den Grad der Versorgung anhand von Luftliniendistanzen, z. T. auch realen Wegedistanzen zwischen Wohn- und nächstem Einkaufsort misst. Der Zugang zu Lebensmitteln wird jedoch über diese Distanz hinaus von weiteren physisch-räumlichen und sozioökonomischen Faktoren beeinflusst. Diese werden bislang kaum berücksichtigt. Ein unzureichender Zugang zu Lebensmitteln ist nicht nur aus gesundheitlicher Perspektive problematisch, sondern v. a. auch aufgrund der sozialen Funktion von Ernährung als wichtigem Feld gesellschaftlicher Teilhabe. Der erschwerte Zugang zu Lebensmitteln trifft insbesondere Menschen in prekären Lebenslagen, die ohnehin bereits in ihrer gesellschaftlichen Teilhabe eingeschränkt sind. In diesem Artikel stellen wir deshalb ein Modell vor, welches theoretisch fundiert die physisch-räumliche und sozioökonomische Einbettung des Lebensmittelzugangs umfassend und systematisch erfasst. Anhand ausgewählter Ergebnisse einer Studie, die mit eben diesem Ansatz in Bremen durchgeführt wurde, zeigen wir, von welchen Zugangsbarrieren Bewohner*innen zweier als gut versorgt geltender Stadtteile betroffen sind. Das vorrangige Ziel dieses Aufsatzes ist es, stärker für die komplexe Problematik des Lebensmittelzugangs zu sensibilisieren. Zudem benennen wir abschließend Handlungsansätze, mit der die Einzelhandelssteuerung und andere kommunale Initiativen den Zugang zu Lebensmitteln verbessern können.

## Abstract (en)

Many German municipalities are developing retail concepts for retail management and taking stock of the food supply is one of the standard tools. A distance-based indicator is commonly used, which measures the degree of supply based on linear distances between the place of residence and the nearest place of purchase. Beyond this distance, however, access to food is influenced by other spatiophysical and socioeconomic factors. So far, these have hardly been considered. Inadequate access to food is not only problematic from a health perspective, but also because of the social function of food as an important field of social participation. Difficult access to food especially affects people in precarious circumstances, who are already restricted in their participation in society. This article therefore presents a model that theoretically informed, comprehensively and systematically captures the spatiophysical and socioeconomic embedding of food access. Based on selected results of a study that was carried out in Bremen using this approach, this article shows the access barriers that affect residents of two districts that are considered to be well supplied. The primary goal of this article is to raise awareness of the complex issue of food access. Finally, approaches that retail and other municipal initiatives can use to improve access to food are also named.

## Einleitung

Die Einzelhandelssteuerung ist ein wichtiges planerisches Betätigungsfeld von Kommunen. Bei der Analyse und Steuerung der Standortwahl des Lebensmitteleinzelhandels dominieren bisher quantitative Ansätze. Diese bewerten den Grad der Versorgung mit Lebensmitteln anhand von Betriebsformenstrukturen, Verkaufsflächengrößen und Luftliniendistanzen, z. T. auch realen Wegedistanzen, zwischen Standorten des Lebensmitteleinzelhandels und Wohngebieten (Jürgens 2014, S. 3). Untersuchungen zur Nahversorgung kommen übereinstimmend zu dem Ergebnis, dass, gemessen an den oben genannten Indikatoren, die Erreichbarkeit des Lebensmitteleinzelhandels v. a. in ländlichen Gebieten unzureichend ist (Bundesministerium für Verkehr, Bau und Stadtentwicklung 2011, S. 20; GfK 2016, S. 1).

Während Versorgungslücken auf dem Land also bereits thematisiert werden, wird den durchaus auch vorhandenen ausgedünnten Versorgungsnetzen in städtischen und großstädtischen Gebieten bisher wenig Aufmerksamkeit geschenkt (Augustin 2020, S. 58). Zudem erfasst die distanzbasierte Untersuchung von Versorgungsqualitäten nur ansatzweise die real vorhandenen Barrieren, die auch in als gut versorgt eingestuften Stadtteilen erlebt werden. Mithilfe von GIS-Analysen können zwar genauere, die Verkehrsinfrastruktur beachtende Analysen durchgeführt werden, die den realen Bedingungen von Einkaufswegen näher kommen (vgl. Burgdorf et al. 2015; Neumeier 2014; Wieland 2015). In der planerischen Praxis auf kommunaler Ebene werden bisher jedoch mehrheitlich Luftliniendistanzen zur Simulation von Entfernungen herangezogen (Augustin 2020, S. 55–56).

Ob in einer Kommune Ernährungssicherheit gegeben ist, kann jedoch mit den rein distanzbasierten Untersuchungsansätzen nicht erhoben werden. Die Welternährungsorganisation der Vereinten Nationen (FAO) definiert Ernährungssicherheit als Zustand, in dem alle Menschen jederzeit ausreichenden physischen und ökonomischen Zugang zu unbedenklichen und nahrhaften Lebensmitteln haben, welche ihren Nährstoffbedarfen und Vorlieben entsprechen und ein aktives und gesundes Leben ermöglichen (Food and Agriculture Organization of the United Nations 1996). Den sozialen, kulturellen und psychologischen Bedeutungen von Ernährung und damit ihrer Relevanz in Bezug auf gesellschaftliche Teilhabe werden die bisherigen Ansätze der Einzelhandelsplanung nicht gerecht. Mit den jüngsten Preisanstiegen für elementare Waren (u. a. Energie und Lebensmittel) erhält das Thema Ernährungssicherheit auch in Deutschland größere Aufmerksamkeit. Obwohl keine amtliche Berichterstattung dazu besteht, ist aus der Armuts- und Ernährungsforschung bekannt, dass insbesondere für armutsgefährdete Haushalte ein erhöhtes Risiko für Ernährungsunsicherheit besteht (Augustin 2020, S. 41).

Notwendig ist also sowohl ein Perspektivenwechsel, welcher die umfassende Bedeutung von Ernährung in den Blick nimmt, als auch eine Rekonzeptualisierung von Zugangsbarrieren. Dazu gehen wir in diesem Artikel zunächst auf die vielfältigen Funktionen von Ernährung ein. Anschließend präsentieren wir ein durch die Erstautorin entwickeltes Modell, welches den Zugang zu Lebensmitteln in seiner physisch-räumlichen und sozioökonomischen Einbettung erfasst. Anschließend zeigen wir anhand einer eigenen empirischen Erhebung, wie mithilfe dieses Modells diverse Zugangsbarrieren in zwei als gut versorgt geltenden Stadtteilen in Bremen aufgedeckt werden können. Auf Grundlage der dargestellten theoretischen Überlegungen und empirischen Erkenntnisse sowie weiterer Literaturanalyse identifizieren wir abschließend Ansatzpunkte und kommunale Handlungsmöglichkeiten zur Verbesserung des Lebensmittelzugangs in Städten.

## Theoretische Einbettung: vielfältige Funktionen von Ernährung

Lebensmittel sichern zunächst das leibliche Überleben und beeinflussen die menschliche Gesundheit. Durch Ernährung im weiteren Sinne werden darüber hinaus jedoch auch soziale und kulturelle Beziehungen gestaltet, und das psychologische Wohlbefinden wird beeinflusst (Feichtinger 1996, S. 7; Heindl 2007, S. 189). Ernährung kann demnach als ein wichtiges Feld gesellschaftlicher Teilhabe angesehen werden (Pfeiffer et al. 2015, S. 6). Das Konzept der gesellschaftlichen Teilhabe beschreibt die Möglichkeit, am Lebensstandard zu partizipieren und Wahl- und Gestaltungsspielräume nutzen zu können, die in einer Gesellschaft in einer konkreten historischen Situation erreicht und angelegt sind (Kronauer und Schmid 2011, S. 160). Teilzuhaben übersetzt sich in der individuellen Erfahrung, mithalten zu können und handlungsmächtig zu sein (Häußermann und Kronauer 2005, S. 17).

Die Wahl des Einkaufsortes, die Auswahl und Zubereitung von Lebensmitteln sowie der Kontext ihres Verzehrs gehören zu den alltäglichen Konsumentscheidungen, in denen Selbst- bzw. Fremdbestimmung erfahren werden. Dies gilt z. B. für die selbstbestimmte Wahl des Geschäftes und den Kauf von Lebensmitteln, die den Vorlieben und gesundheitlichen, sozialen und kulturellen Bedürfnissen des Haushaltes entsprechen. Können Einkaufsort und die Auswahl der Lebensmittel regelhaft nicht selbstbestimmt gewählt werden, weil physisch-räumliche bzw. sozioökonomische Bedingungen der Wahl entgegenstehen, kann dies als gesellschaftlicher Ausschluss erfahren werden. Dies ist von besonderer Bedeutung, wenn Teilhabemöglichkeiten in anderen Bereichen, z. B. über das Arbeitsleben, entfallen. Dies ist beispielsweise bei prekären Beschäftigungsverhältnissen der Fall, die weniger Möglichkeiten für selbstbestimmtes Handeln bieten. Insbesondere für marginalisierte gesellschaftliche Gruppen kann Konsum somit ein wichtiges Feld bilden, in dem Identität demonstriert und Handlungsfähigkeit erfahren werden kann (Bosch 2010, S. 462).

## Ein umfassendes Zugangskonzept zur Anerkennung der vielfältigen Funktionen von Ernährung

Die Wahl der Einkaufsstätte gehört zu den in der geografischen Konsumforschung am längsten untersuchten Fragestellungen (Popp 2020, S. 76). Die Nähe zur Einkaufsstätte wird dabei als wichtiger, aber keineswegs einziger Faktor für die Wahl der Einkaufsstätte herausgearbeitet (Wieland 2021, S. 118). Wie wir andernorts entsprechend herausgearbeitet und ausführlicher dargestellt haben (Augustin 2020, S. 88–93), ist der Zugang zu Lebensmitteln multidimensional zu erklären. Erstens wird der Zugang zu Lebensmitteln von physisch-räumlichen Faktoren beeinflusst. Dazu zählen die Standortverteilung des Lebensmitteleinzelhandels, die lokale Auswahl und die Preise und Qualität der angebotenen Lebensmittel. Auch der Grad der Barrierefreiheit der Einkaufswege zählt zu diesen physisch-räumlichen Faktoren. Zweitens beeinflussen sozioökonomische Situation, Motive, Einstellungen und Werte eines Haushalts den Zugang zu Lebensmitteln. Die sozioökonomische Situation ist davon gekennzeichnet, über welche gesellschaftlich relevanten Ressourcen, z. B. Einkommen, Vermögen, Bildungsabschlüsse und Kompetenzen der Haushalt verfügt.

**Abb. 1:**
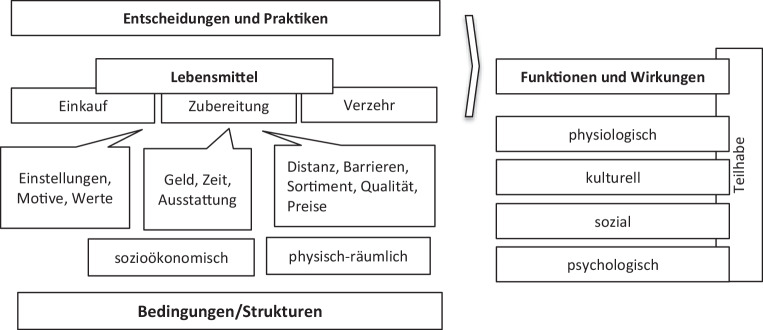
Bedingungen und Wirkungen von Ernährungspraktiken. (Quelle: Hanna Augustin CC BY-NC-SA)

**Abb. 2:**
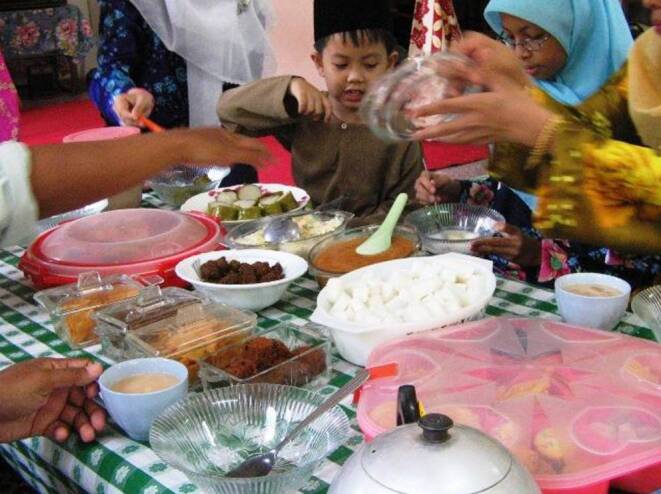
Mahlzeit: Gelegenheit zur Pflege sozialer Beziehungen. (Quelle: Syefri Zulkefli from Shah Alam, Malaysia, CC BY-SA 2.0)

Modellhaft lässt sich die Einbettung des Zugangs zu Lebensmitteln in den physisch-räumlichen Gegebenheiten und in den sozioökonomischen Strukturen wie folgt darstellen:

Die Abb. 1 stellt den Zusammenhang her zwischen den Bedingungen, unter denen Lebensmittel erworben, zubereitet und verzehrt werden, und den Wirkungen, die der Einkauf, die Zubereitung und die Form des Verzehrs von Lebensmitteln unter den gegebenen Umständen entfalten. In Bezug auf die Wirkungsbereiche ergänzen wir die Ernährungssicherheitsdefinition der FAO um die sozialen, kulturellen und psychischen Funktionen von Ernährung: Über das Essen und Trinken werden Nährstoffe aufgenommen (physiologische Funktion), aber auch soziale Beziehungen gepflegt, Zugehörigkeit und Nähe gefördert (soziale und psychische Funktion) (s. Abb. 2). Über die Auswahl oder die Art der Zubereitung von Lebensmitteln können außerdem religiöse oder moralische Überzeugungen ausgedrückt werden (kulturelle Funktion) (Feichtinger 1995, S. 292–293; Wahl und Schulte 2011, S. 373). Die sozioökonomischen und physisch-räumlichen Bedingungen des Lebensmittelzugangs spannen demnach einen spezifischen Handlungsrahmen für den Einkauf, die Zubereitung und die Gestaltung der Verzehrsituation auf. Ein restriktiver Handlungsrahmen kann zu multiplen Einschränkungen der mit Ernährung verbundenen Funktionen führen.

Wir erkennen an, dass die Ursachen sozioökonomischer Barrieren, d. h. soziale Ungleichheit, kaum planerisch beeinflusst werden können. Dennoch meinen wir, dass die Planung bzw. kommunale Politik stärker als bisher zur Verbesserung des Lebensmittelzugangs beitragen kann. Voraussetzung dafür ist, dass zunächst anerkannt wird, dass der Zugang von sehr unterschiedlichen Faktoren auf verschiedenen Ebenen beeinflusst wird. Untersuchungen und Ansätze sollten entsprechend so umfassend wie möglich gestaltet werden, um Barrieren aufdecken, Handlungsoptionen zu identifizieren und letztlich die Erreichbarkeit von Lebensmitteln zu verbessern. Mit diesem Beitrag möchten wir den geforderten Perspektivenwandel theoretisch unterlegen und anhand eines empirischen Beispiels ausführen und unterstützen.

## Fallstudie zu Zugangsbarrieren in Bremen: Methoden

Wie beschrieben, wird in der kommunalen Planung bisher der Grad der Versorgung mit Lebensmitteln über Untersuchungen der Betriebsformenstrukturen, Verkaufsflächen und Luftlinien- oder realen Wegedistanzen zwischen Standorten des Lebensmitteleinzelhandels und Wohngebieten beschrieben (vgl. Acocella et al. 2012, 2014; Freie Hansestadt Bremen, Die Senatorin für Klimaschutz, Umwelt, Mobilität und Stadtentwicklung und Die Senatorin für Wirtschaft, Arbeit und Europa 2020). Die Bewertung der Versorgung erfolgt demnach ausschließlich anhand des Lebensmittelhandels und ohne die Perspektive der lokalen Bevölkerung einzubeziehen. Als gut versorgt gelten Stadtteile, in denen ein großer Teil der Bevölkerung innerhalb eines bestimmten Radius, meist zwischen 500 und 700 m, um einen Super- oder Verbrauchermarkt wohnt. Eine Distanz von 500–700 m wird als fußläufig erreichbar angenommen.

**Abb. 3:**
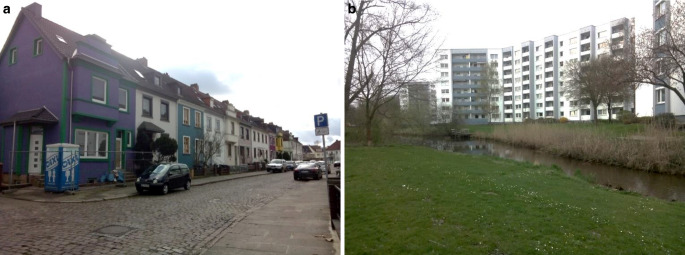
**a**, **b** Bebauungsstrukturen in Gröpelingen (**a**) und der Vahr (**b**). (Foto: Augustin 2016, CC BY-NC-SA)

Unsere im Rahmen einer Dissertation 2016 durchgeführte Untersuchung stellte jedoch fest, dass dieser Indikator wichtige weitere räumlich-physische Barrieren unterschlägt und sozioökonomische Barrieren nicht erfasst. Wir untersuchten die Bremer Stadtteile Gröpelingen (rd. 37.500 Einwohner*innen) und Vahr (rd. 27.200 Einwohner*innen). Beide Stadtteile sind im gesamtstädtischen Vergleich dicht besiedelt und sind Wohnort vieler Menschen, die unter schwierigen sozioökonomischen Bedingungen leben (Augustin 2020, S. 119–120). Von der Siedlungsstruktur unterscheiden sich die beiden Stadtteile jedoch deutlich: Während Gröpelingen als hafennahes, gewachsenes Arbeiter*innenquartier zu sehen ist, wurden die Großwohnsiedlungen der Vahr in den 1960er Jahren nach dem Ideal der autogerechten Stadt entworfen (s. Abb. 3a, b). Die beiden Stadtteile stehen damit exemplarisch für 2 baustrukturelle Typen, die in vielen deutschen Städten die Wohnlagen prekärer Bevölkerungsgruppen bilden (Farwick 2007, S. 44), was ihre Auswahl für unsere Untersuchung begründet.

Um Hürden im Zugang zu Lebensmitteln aufzudecken, wurden geografische Methoden mit quantitativen und qualitativen Ansätzen der empirischen Sozialforschung verknüpft. Zunächst wurden mittels einer Kartierung aller lebensmittelverkaufenden Geschäfte in den Stadtteilen Gröpelingen und Vahr die räumliche Verteilung von Versorgungsstandorten erhoben. Da die Kartierung auch das Angebot an frischem Obst und Gemüse umfasste, konnte außerdem eine Übersicht über das vorhandene Lebensmittelangebot erworben werden.

Anschließend wurden auf Basis eines theoretischen Samplings ausgewählte Bewohner*innen Gröpelingens und der Neuen Vahr in Einkaufszentren, Freizeit‑, Kultur- und Beratungseinrichtungen und Ausgabestellen der Bremer Tafel befragt (*N* = 125). Die Auswahl der Befragungsorte orientierte sich an den zu untersuchenden Benachteiligungsvariablen, die im Sample vertreten sein sollten (z. B. Armut, Migrationshintergrund, eingeschränkte Mobilität). Die Befragungsstichprobe spiegelt den im gesamtstädtischen Vergleich überdurchschnittlichen Anteil an Bewohner*innen in prekären Lebenslagen in beiden Stadtteilen wider. Mit der ausgewählten Stichprobe können Zusammenhangshypothesen zwischen sozialstrukturellen Variablen (z. B. Geschlecht und Klasse) und Einschränkungen im Zugang zu Lebensmitteln überprüft werden.

Zugangsbarrieren wurden in der Befragung über 8 Kriterien erfasst, die u. a. erfragen, wie viel Zeit für Einkaufswege aufgewendet wird, ob physisch-räumliche Hindernisse auf den Wegen bestehen, aber auch wie die Zufriedenheit mit dem Sortiment und der Lebensmittelqualität ausgeprägt ist (s. Tab. 1). Um Zusammenhänge zwischen sozioökonomischen Lebensbedingungen und Zugangsbarrieren zu erkennen, wurden die Befragungsdaten in Kreuztabellen und mit auf Chi^2^ basierenden Zusammenhangsmaßen ausgewertet.

**Tab. 1:** Variablen zu Einschränkung im Zugang zu Lebensmitteln in Gröpelingen und der Vahr. (Quelle: Augustin 2020, S. 162 und 175)

	Nein (Keine Einschränkung) [%]	Ja (Einschränkung) [%]
Dauert der Einkaufsweg zu häufig aufgesuchten Geschäften länger als 10 min? (*N* = 122)	51,6	48,4
Gibt es Hindernisse auf dem Einkaufsweg? (*N* = 88)	71,6	28,4
Werden Besuche von bestimmten Geschäften durch Hindernisse im Straßenraum verhindert? (*N* = 78)	76,9	23,1
Ist der Einkaufsweg mäßig bis sehr anstrengend? (*N* = 89)	74,2	25,8
Besteht volle Zufriedenheit mit dem vorhandenen Sortiment? (*N* = 122)	85,2	14,7
Besteht volle Zufriedenheit mit der vorhandenen Lebensmittelqualität? (*N* = 121)	50,2	48,8
Sind alle benötigten Lebensmittel zu erschwinglichen Preisen erhältlich? (*N* = 117)	82,9	17,1
Werden neben Discountern auch Geschäfte anderer Betriebsformen aufgesucht? (*N* = 124)	87,1	12,9

Darüber hinaus wurden qualitative Interviews mit zentralen Akteuren in der kommunalen Verwaltung, der Standortplanung wichtiger Lebensmittelhandelsketten sowie mit Sozialarbeiter*innen in den beiden Stadtteilen geführt. Das Interviewmaterial wurde gemäß der inhaltlich strukturierenden qualitativen Inhaltsanalyse nach Kuckartz (Kuckartz 2012) ausgewertet.

## Fallstudie zu Zugangsbarrieren in Bremen: Ergebnisse

In Gröpelingen und der Vahr bilden Verbrauchermärkte, Supermärkte und kleinflächige Lebensmittelmärkte (sowohl mit deutschem Standardsortiment als auch spezialisiert auf die arabische, türkische oder osteuropäische Küche), Discounter, Fachgeschäfte wie Bäckereien und Metzgereien, Feinkostläden und Kioske mit Lebensmittelsortiment die lokalen Einkaufsstrukturen. Während in Gröpelingen in vielen Geschäften Lebensmittel angeboten werden, die insbesondere in der türkisch-arabischen Küche Verwendung finden, ist in der Vahr eine Spezialisierung auf Zutaten für die osteuropäische Küche festzustellen. Gemäß distanzbasierten Kriterien und im Vergleich zu anderen Großstädten gelten beide Gebiete als gut versorgt: Nur rund 10 % der Stadtteilbevölkerung wohnen weiter als 600 m Luftlinie vom nächsten Vollsortimentsgeschäft entfernt. Wie unsere Untersuchung jedoch zeigt, erfahren Bewohner*innen nichtsdestotrotz Barrieren im Zugang zu Lebensmitteln. Sowohl hinsichtlich ihrer Gesundheit als auch in Bezug auf gesellschaftliche Teilhabe über Lebensmittel und Ernährung erscheint dies bedenklich.

Barrieren bestehen in den untersuchten Stadtteilen erstens in Bezug auf die Einkaufswege und zweitens in Bezug auf das Sortiment und die Möglichkeiten seiner Nutzung (s. Tab. 1).

Im Folgenden zeigen wir für einige der in der Tabelle gelisteten Einschränkungen auf, wie sozioökonomische und physisch-räumliche Faktoren zusammenspielen und zu unterschiedlichen Betroffenheiten innerhalb der Stadtteilbevölkerung führen.

Die Einkaufswege sind für die Befragten in unterschiedlichem Ausmaß geprägt durch die benötigte Wegezeit sowie durch Hindernisse und Anstrengung, die auf den Wegen erlebt werden. So benötigt rund die Hälfte der Befragten mehr als 10 min für die Einkaufswege zu wöchentlich aufgesuchten Geschäften. Gemäß der gemeinhin verwendeten Definition gilt eine Entfernung, deren Bewältigung länger als 10 min kostet, nicht mehr als fußläufig. Wie in Planer*innenkreisen bereits bekannt und durch die aktionsräumlichen Ansätze der geografischen Handelsforschung aufgegriffen, kann an dieser Stelle erneut bestätigt werden, dass Konsument*innen keineswegs stets die nächstgelegene Einkaufsgelegenheit wahrnehmen, sondern für die für sie aus unterschiedlichen Gründen passende Einkaufsgelegenheit ggf. längere Einkaufswege in Kauf nehmen (Popp 2020, S. 77–79).

Von dieser Barriere besonders betroffen sind in unserer Untersuchung mobilitätseingeschränkte Personen, die ihre Kochweise, Rezepte und verwendete Zutaten als „deutsch“ beschreiben. Mobilitätseingeschränkte Personen, die ihre Ernährung beschreiben als beeinflusst von z. B. türkischer oder russischer Küche, sprechen dieses Hindernis nicht in gleichem Maße an. Dieses Ergebnis weist darauf hin, dass die zweite genannte Gruppe die lokale Einzelhandelsstruktur umfänglicher nutzt und beispielsweise auch kleinflächige Märkte nutzt, deren Angebot vom deutschen Standardsortiment abweicht. Rund ein Viertel der Befragten benennt physische Barrieren, die den Einkaufsweg erschweren: Zu weite Distanzen spielen dabei jedoch eine untergeordnete Rolle. Stattdessen wird in unserer Untersuchung v. a. der Zustand von Straßen und Wegen als Hindernisse benannt, der die Fortbewegung mit Rollatoren und Rollstühlen erschwert.

**Abb. 4:**
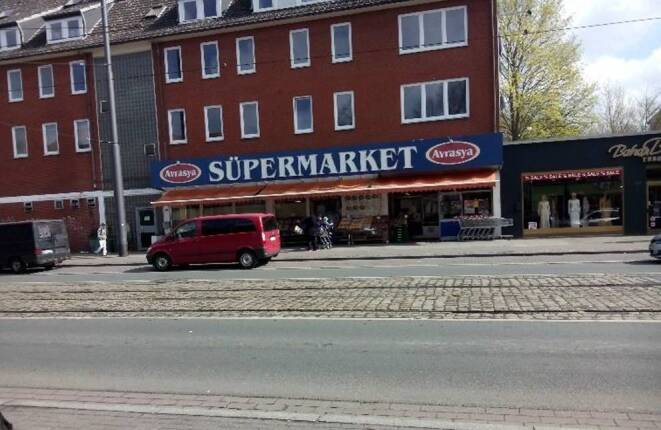
Gleisbett in Gröpelingen erschwert Straßenquerung mit Rollstuhl und Rollator. (Foto: Augustin 2016, CC BY-NC-SA)

**Abb. 5:**
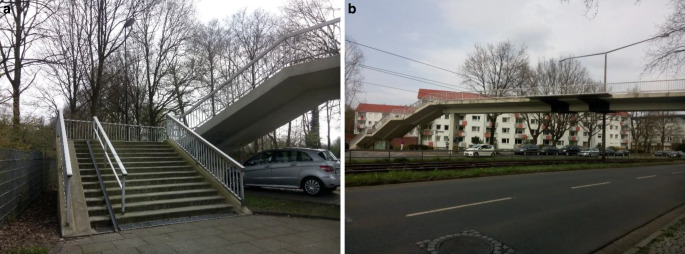
**a**, **b** Fußgängerüberführung in der Vahr als städtebauliche Barriere. (Foto: Augustin 2016 CC BY-NC-SA)

Als hinderlich werden beispielsweise Kieswege, fehlende Bordsteinabsenkungen, Straßenbahngleise (s. Abb. 4) und durchwurzelte Gehwegplatten angegeben. Auch als unzureichend empfundene Querungsmöglichkeiten von Straßen gehören zu den am meisten genannten Hindernissen (s. Abb. 5a, b); 23 % der Befragten berichten, dass es Lebensmittelgeschäfte gibt, die sie gerne aufsuchen würden, deren Entfernung vom Wohnort dies jedoch verhindere; 26 % der Befragten empfinden ihre Einkaufswege als anstrengend. Die statistische Analyse zeigte, dass dies mit größerer Wahrscheinlichkeit bei Befragten vorkommt, die unter der Armutsgefährdungsschwelle leben (Augustin 2020, S. 168–172).

Die im Sample am weitesten verbreitete Einschränkung bezüglich des Angebotes betrifft die Wahrnehmung der angebotenen Lebensmittelqualität. Etwa die Hälfte der Befragten ist mit dieser nicht vollkommen zufrieden. Marktforschungsstudien bestätigend sind Befragte mit hohem Bildungsabschluss dabei weniger zufrieden als Befragte mit niedrigem Bildungsabschluss. Ein bisher nicht beschriebener Zusammenhang besteht zwischen der Versorgung bei Lebensmitteltafeln und der Bewertung der im Handel angebotenen Lebensmittelqualität: Personen, die sich auch über Lebensmitteltafeln versorgen, sind zufriedener mit der Lebensmittelqualität des Lebensmitteleinzelhandels als Befragte, die keine Lebensmitteltafel besuchen. Die Qualität der bei der Tafel ausgegebenen Waren lässt die Lebensmittel im Handel offensichtlich in günstigerem Licht erscheinen. Andere, auf das lokale Lebensmittelangebot bezogene Einschränkungen wie wahrgenommene Sortimentslücken, unzureichende finanzielle Ressourcen oder eine auf Discounter beschränkte Nutzung der Lebensmittelinfrastruktur betreffen zwischen 13 % und 17 % der Befragten.

Die Ergebnisse zu lebensmittelbezogenen Einschränkungserfahrungen weisen vorwiegend Zusammenhänge zu klassenbezogenen Faktoren auf: Studien aus der soziologischen Armutsforschung (Chassé et al. 2007, S. 117–119; vgl. Lehmkühler 2002; vgl. Pfeiffer et al. 2015; Statistisches Bundesamt 2021, S. 29) bestätigend, zeigt unsere Untersuchung in Bremen, dass ein Leben in Armut auch mit Entbehrungen bei der Ernährung verbunden ist. Ausschließlich im Discounter versorgen sich insbesondere Befragte, die auch eine Lebensmitteltafel besuchen. Dieses Ergebnis verdeutlicht, dass auch im wohlhabenden Deutschland Bevölkerungsgruppen darauf angewiesen sind, ihre Ernährung aus einer Kombination von (fast) kostenlosen und sehr günstigen Lebensmitteln zusammenzustellen. Dabei muss berücksichtigt werden, dass auch hier die Kochtradition moderierend wirkt: Tafelnutzer*innen, die nicht nur deutsche Rezepte und Zutaten verwenden, versorgen sich mit geringerer Wahrscheinlichkeit ausschließlich bei Discountern als diejenigen, die ihre Ernährung als „deutsch“ beschreiben.

Die Bremer Fallstudie zeigt insgesamt, dass Zugangsbarrieren physisch-räumlicher und sozioökonomischer Art auch in Stadtteilen bestehen, die als gut versorgt gelten. In Bezug auf die physisch-räumlichen Faktoren stellten wir zunächst fest, dass der Luftlinienindikator, mit dem die Wege zwischen Wohn- und Einkaufsadresse simuliert werden, nur wenig aussagekräftig ist: Fast die Hälfte der Befragten benötiget länger als 10 min für den Weg zu häufig aufgesuchten Lebensmittelgeschäften. Neben Distanz und Wegedauer, welche den Lebensmittelzugang beeinflussen, stellt sich u. a. die Wegebeschaffenheit z. T. als Hindernis dar. Zweitens konnten wir aufzeigen, wie komplexe sozioökonomische Positionen auf den Lebensmittelzugang wirken: In der gleichen Einkaufsumgebung erfahren von Armut betroffene und/oder mobilitätseingeschränkte Bewohner*innen häufiger und andere Einschränkungen. Für Personen mit Migrationshintergrund oder multinationaler Kochtradition sind die beschriebenen Zusammenhänge zwischen Armut, Mobilität und Zugangsbarrieren interessanterweise weniger stark ausgeprägt. Wie erwähnt, könnte eine umfänglichere Nutzung der lokalen Einzelhandelsstruktur, auch beispielsweise der kleinflächigen Märkte, deren Angebot vom deutschen Standardsortiment abweicht, hierfür eine Erklärung sein.

## Ausblick

Abschließend möchten wir einige aus unserer Untersuchung sowie einer Literaturauswertung resultierende Empfehlungen und Handlungsoptionen für Kommunen skizzieren.

- Grundsätzlich gilt, dass der Lebensmittelzugang als* integratives kommunales Handlungsfeld *verstanden werden sollte, welche nicht nur der Gesundheit und dem Wohlbefinden der Bewohner*innen dient, sondern auch gesellschaftliche Teilhabe fördern kann.
- Um Lebensmittelversorgung möglichst umfassend und für alle gesellschaftlichen Gruppen zu ermöglichen, ist eine *Zusammenarbeit über Verwaltungsressortgrenzen* hinweg nötig. Zudem bietet es sich an, auch lokale Akteure aus dem Bereich der Gemeinwesenarbeit oder sozialen Arbeit auf Stadtteilebene einzubeziehen.
- Dabei darf die *Verantwortung* für Ernährungssicherung *nicht* auf *zivilgesellschaftliche Initiativen* z. B. Urban-gardening-Gruppen abgewälzt werden. Denn „(…) bei aller Wertschätzung der Gärten und Gärtnernden (…) [muss] vor einer Verklärung urbaner Kleinstlandwirtschaft gewarnt werden. Denn ebenso wenig wie Gemeinschaftsgärten als Ersatz für öffentliche Grünflächen geeignet sind, dürfen sie als Ersatz für eine soziale Grundsicherung gelten.“ (Rosol 2014, S. 224)
- Im Bewusstsein für die Bedeutung des Lebensmitteleinkaufs – inklusive seiner Bedeutung als Feld gesellschaftlicher Teilhabe – könnte die *Einzelhandelssteuerung* in den Kommunen einen wesentlich größeren Beitrag zur Analyse des Problemfeldes leisten und damit den Grundstein zur Verbesserung der Situation legen. Ein erster Schritt wäre die Anpassung der Erhebungen des Lebensmitteleinzelhandels zu einer systematischen Erfassung des Lebensmittelzugangs in all seinen Dimensionen, d. h. die Schaffung einer soliden Datenbasis zu sozioökonomischen Barrieren und über Distanzen hinausgehende physisch-räumliche Gegebenheiten. Dazu sollte zunächst eine Sensibilität dafür entwickelt werden, dass der Zugang zu Lebensmitteln nicht allein von physisch-räumlichen Faktoren, insbesondere nicht ausschließlich durch die Distanz zwischen Wohn- und Einkaufsort geprägt wird. Unser Modell liefert dafür eine Grundlage. Gegebenenfalls kann der Arbeitsaufwand, der für die Erarbeitung einer soliden Datenbasis nötig ist, durch die Zusammenarbeit mit Hochschulen gemindert werden.
- Neben der bereits angesprochenen Steuerung von Einzelhandelsstrukturen gibt es auch Handlungsspielräume bei der *Straßenraumgestaltung*, welche klar in der Hand der Kommune liegt. Wie unsere Untersuchung deutlich gezeigt hat, erleichtert eine barrierearme Straßenraumgestaltung den Zugang zu Lebensmitteln.
- Darüber hinaus können in einem gewissen Umfang auch *sozioökonomische Bedingungen* des Lebensmittelzugangs beeinflusst werden. Obgleich deren Ursprung auf gesamtgesellschaftlicher Ebene zu verorten ist, gibt es auch auf kommunaler Ebene Handlungsoptionen, um Ausgaben für zentrale Grundbedürfnisse zu reduzieren sowie Haushaltseinkommen zu erhöhen. Dazu gehört die öffentliche und kostengünstige Bereitstellung von zentralen Elementen der *Daseinsvorsorge*, insbesondere über den kommunalen Wohnungsbau und entsprechende Mietpreispolitik. Auf der anderen Seite können kommunale Arbeitsmarkt- und Fördermaßnahmen bzw. Wirtschafts- und Sozialpolitik im weiteren Sinne zur Steigerung von Einkommen beitragen.
- Die untersuchten Stadtteile gelten auch deshalb als gut versorgt, da sich neben den Geschäften der großen Handelsketten, die den stark konzentrierten Lebensmitteleinzelhandel dominieren, (noch) kleinere, nicht-filialisierte Märkte halten können, die die Nahversorgung sichern. Die explizite *Förderung* dieser *Kleinteiligkeit*, womöglich auch gemeinwohlorientierter Angebote, wäre eine weitere Möglichkeit, fußläufige Nahversorgung zu stärken. Dabei stellt sich auch die Frage, ob Städte einen stärkeren Einfluss auf das Sortiment der Geschäfte nehmen wollen, um das Angebot an unverarbeiteten pflanzlichen Lebensmitteln zu erhöhen.
- Auf kommunaler Ebene zu Ernährungssicherheit beizutragen ist keinesfalls als alleinige Aufgabe der Einzelhandelsplanung zu verstehen. Städtische Ernährungspolitik und -planung, die Verwaltungsgrenzen überwindet, bietet ein großes Potenzial für eine nachhaltige Stadtentwicklung, sei es in den Bereichen Verkehr und Logistik, lokale Wirtschaft, Abfallwirtschaft oder Bildung (Koç et al. 1999; Pothukuchi und Kaufman 1999; Mendes 2017; Sonnino et al. 2019). Anregungen für eine *kommunale Ernährungspolitik* bietet der *Milan Urban Food Policy Pact* (MUFPP 2015), der weltweit seit 2015 bereits von über 200 Städten (darunter in Deutschland Berlin, Frankfurt und Köln) unterzeichnet wurde. Das MUFPP ist ein umfassendes internationales Abkommen von Bürgermeister*innen, welches eine Vielzahl von ernährungspolitischen Herausforderungen sowie die besondere Rolle der Städte bei deren Bewältigung explizit auflistet. Die unterzeichnenden Städte verpflichten sich, sowohl auf Gerechtigkeit als auch auf Nachhaltigkeit hinzuarbeiten (MUFPP 2015, S. 1.).
